# Steroid concentrations in antepartum and postpartum saliva: normative values in women and correlations with serum

**DOI:** 10.1186/2042-6410-4-7

**Published:** 2013-04-10

**Authors:** Elizabeth Hampson, Shauna-Dae Phillips, Claudio N Soares, Meir Steiner

**Affiliations:** 1 Department of Psychology and Graduate Program in Neuroscience, University of Western Ontario, London, ON, Canada; 2 Women’s Health Concerns Clinic, St. Joseph’s Healthcare; Department of Psychiatry and Behavioural Neurosciences and Obstetrics & Gynecology, McMaster University, Hamilton, ON, Canada

**Keywords:** Saliva, Steroid, Enzyme immunoassay, Radioimmunoassay, Pregnancy, Postpartum, Gestation, Hormone

## Abstract

**Background:**

Saliva has been advocated as an alternative to serum or plasma for steroid monitoring. Little normative information is available concerning expected concentrations of the major reproductive steroids in saliva during pregnancy and the extended postpartum.

**Methods:**

Matched serum and saliva specimens controlled for time of day and collected less than 30 minutes apart were obtained in 28 women with normal singleton pregnancies between 32 and 38 weeks of gestation and in 43 women during the first six months postpartum. Concentrations of six steroids (estriol, estradiol, progesterone, testosterone, cortisol, dehydroepiandrosterone) were quantified in saliva by enzyme immunoassay.

**Results:**

For most of the steroids examined, concentrations in antepartum saliva showed linear increases near end of gestation, suggesting an increase in the bioavailable hormone component. Observed concentrations were in agreement with the limited data available from previous reports. Modal concentrations of the ovarian steroids were undetectable in postpartum saliva and, when detectable in individual women, approximated early follicular phase values. Only low to moderate correlations between the serum and salivary concentrations were found, suggesting that during the peripartum period saliva provides information that is not redundant to serum.

**Conclusions:**

Low correlations in the late antepartum may be due to differential rates of change in the total and bioavailable fractions of the circulating steroid in the final weeks of the third trimester as a consequence of dynamic changes in carrier proteins such as corticosteroid binding globulin.

## Background

Saliva has been advocated as an alternative to serum or plasma for the measurement of steroid hormones, and offers a significant theoretical advantage as a diagnostic fluid: hormone concentrations in saliva derive primarily from free steroid present in the general circulation [1-4], whereas steroid bound to high-affinity binding proteins such as sex-hormone binding globulin (SHBG) or corticosteroid-binding globulin (CBG) is present in saliva at levels only ~0.1% of the levels seen in serum or plasma [5,6]. Accordingly, steroid concentrations in saliva most closely approximate the fraction of the steroid in circulation that is ‘bioavailable’, i.e., that fraction of the total hormone that is able to exert physiological effects. The salivary glands contain the enzyme 11β-hydroxysteroid dehydrogenase II and therefore, for cortisol, the glands do metabolize some active hormone to cortisone *en passant*[4]. Even for cortisol, however, concentrations in saliva have been found to correlate highly (*r* = .95 or higher) with the free fraction measured in serum. At present, there is no simple, accurate, and cost-effective method to isolate this physiologically important fraction from blood, making saliva an attractive alternative for many research and clinical applications.

Accurate quantification of hormones in saliva requires assays with greater sensitivity and precision than those developed for blood, because under normal conditions only a small fraction of the steroid (1 - 8%, depending on the hormone) circulates in unbound form. Most published validity studies examining the correlations between saliva and serum total or serum free steroid concentrations are based on radioimmunoassay (RIA) techniques, but viable non-isotopic methods are starting to become commercially available for saliva. The fact that correlations approaching *r* = 1.0 have been demonstrated for cortisol, progesterone, testosterone, estriol, and even estradiol in some studies (e.g., [7-12]; for review see [2,13]) supports the basic validity of salivary techniques. The magnitude of the correlations, however, and thus the relative utility of saliva *versus* blood, can be influenced by a number of methodological and demographic variables, not all of which have been identified. The correlations depend, for instance, on the particular hormone analyzed, the subject population, type of assay used, assay parameters such as sensitivity or specificity of the antiserum, whether saliva is compared with the free or total fraction in plasma, and adequacy of the saliva collection method. Proper collection and storage are important [14-16].

Saliva has potential clinical applications in maternal health monitoring. One impediment to its adoption, however, is the limited data currently available on the use of saliva to measure steroids during pregnancy and the postpartum. Normative data for a range of salivary steroids during pregnancy are not yet available, especially for the sex steroids (*cf*., [17]). Even fewer studies have examined the *correlations* between serum and saliva during pregnancy, and thus the viability of saliva as a substitute for serum determinations. Pregnancy and post-partum represent the maximal and minimal expected hormone concentrations that occur in women of reproductive age and may pose a challenge to salivary assays which generally are optimized to detect differences within the concentration ranges defined by the menstrual cycle. Significant changes in carrier proteins, including SHBG and CBG, also occur during pregnancy and could potentially alter the size of the correlations between serum and saliva.

Outside of pregnancy, cortisol is considered the best-validated salivary analyte [18], but estriol is perhaps the best validated analyte in pregnant women. Salivary monitoring of estriol concentrations has been suggested as a way to assess fetoplacental function. Early work using RIA reported an excellent correlation between saliva and either free estriol or total estriol in serum (r = 0.98 [8,19,20]; for reports using enzyme immunoassay (EIA) r = 0.87 [21], r = 0.79 [22]). Pregnancy and the postpartum are characterized by large changes in cortisol production, CBG levels, and responsivity of the hypothalamic-pituitary-adrenal axis [23,24], but scant attention has been paid to serum-saliva correlations during the reproductive period. Dorn and Susman [25] found that at ≤ 20 weeks gestation, salivary cortisol showed relatively low correlations with total cortisol in serum, ranging from 0.72 to 0.77. Correlations at 2–3 weeks postpartum were even lower (r = 0.42 to 0.60). It is unknown whether or not such low correlations are typical of the postpartum period. These correlations are lower than those observed for salivary cortisol in many other contexts (e.g., r = 0.97 [10,26,27]) and, if verified, could conceivably reflect latent changes in binding proteins during pregnancy. Lowered correlations between saliva and serum total cortisol concentration have been found previously in situations where there is significant patient-to-patient variation in CBG levels or in CBG saturation due to medication use (e.g., oral estrogens) or other factors (e.g., r = 0.67 [28]; r = 0.60 [29]). In such situations the tight linear association between saliva and the serum *unbound* fraction is faithfully preserved [10].

Other salivary steroids have received negligible investigation in the context of pregnancy. Serum estradiol rises steadily toward term, but the expected concentrations for salivary estradiol are unknown, as is the serum-saliva correlation during pregnancy. Changes in SHBG titers could potentially reduce the correlation between the bioavailable fraction, mirrored in saliva, and total estradiol as measured in serum. In the postpartum, extremely low estradiol secretion is a further complication that poses a technical challenge for accurate quantification. In saliva, the expected levels of estradiol over the normal menstrual cycle are in the low picomolar or femtomolar range and, while satisfactory correlations can be seen under relatively high estradiol conditions (e.g., *in vitro* fertilization with ovarian stimulation, r = 0.86 [30]; r = 0.82 [31]; r = 0.94 [12]), variable correlations have been found over the ordinary menstrual cycle (r = 0.83 [32]; r = 0.93 [33]) with lower correlations in some studies (r = .60 to .80) reflecting, among other things, difficulty in establishing satisfactory assay precision at the low end of the concentration range (*cf.*, [34]). Technical challenges are likely to be accentuated in the postpartum. For progesterone, the correlation between saliva and serum in healthy women typically ranges from r = 0.80 to 1.0 (r = 0.91 to 0.97 [35]; r = 0.93 [36]; r = 0.84 to 0.89 [37]; r = 0.85 [38]; r = 0.88 [39]; r = 0.98 [7]). Although only limited data are presently available for progesterone during pregnancy, estimates of the serum-saliva correlation are inconsistent and differ widely across studies (e.g., r = 0.46 [40]; r = 0.88 [41]).

Salivary testosterone presents special issues. In men, salivary testosterone closely mirrors the free fraction of testosterone in the serum (r = 0.80 to 0.97 [11,42,43]) but in women, the adequacy of salivary testosterone measures has been more difficult to establish and correlations reported are often only moderate in size (e.g., r = 0.55 to 0.85 [11,44-49]). Inadequate antibody specificity or analytical sensitivities in some studies may potentially explain the lower correlations, but failure to take availability of binding proteins into account may also contribute; many validation studies have included not just healthy controls but also women with hirsutism, polycystic ovarian disease, or obesity, conditions that alter levels of SHBG. Judicious choice of the *serum* assay used is important but seldom considered--many commercial immunoassays for testosterone significantly underperform if applied to female serum [50].

The present study investigated the associations between salivary and serum measures of six steroids (estradiol, estriol, progesterone, testosterone, cortisol, DHEA) in a group of women who donated paired saliva and serum specimens during the third trimester of pregnancy or the first six postpartum months. This work is a first step toward establishing the validity of saliva as an alternative to blood for steroid monitoring during pregnancy, and to establishing normative ranges for saliva during pregnancy and the postpartum using a widely available commercial EIA.

## Methods

### Participants

Data were available from 28 women tested between 32 and 38 weeks of gestation and 43 women who were tested between 4 and 16 weeks postpartum (*n* = 30) or at 19+ weeks (*n* = 13). Women were recruited from a hospital perinatal or obstetric clinic or from local perinatal fitness classes, and consented to the collection of serum and saliva as part of a broad study of mood and memory in the peripartum, which was approved by the St. Joseph’s Healthcare Research Ethics Board. The specific time frames sampled were dictated by the parent study, as probable times to capture peripartal changes in mood. Mean age was 30.32 (± 5.05 yr). All pregnancies were full term and uncomplicated. Most were primiparous (74%). As part of a broader study protocol, the presence of mood disorders was formally evaluated. Ten women met DSM-IV criteria for major depression according to the Mini International Neuropsychiatric Interview and scored ≥ 11 on the Edinburgh Postnatal Depression Scale (EPDS). Of the 43 postpartum women, three met the criteria for depression. All remaining women were healthy, scored <11 on the EPDS and had no current or past history of psychiatric or chronic medical conditions or of substance/alcohol use. Oral contraceptive use in the postpartum was screened and data from one woman were excluded on that basis.

### Sample collection

All specimens were collected at a fixed time of day (1200±2 hrs). Because several of the steroids of interest show a circadian pattern of release, midday sampling was chosen as a period of relative stability in the concentrations, in order to avoid early morning or evening sampling when the concentrations of several hormones undergo rapid changes that could potentially attenuate the correlations observed between serum and saliva. Each participant provided one saliva sample and a matched sample of serum. Saliva was collected via passive drool into a 15 mL plain polystyrene tube. Participants were asked to refrain from eating, drinking, smoking, brushing their teeth, chewing gum or other oral stimulation for 1 hr prior to sample collection. Participants rinsed their mouths, waited 5 min, then donated 3 mL of saliva. The saliva was divided into separate aliquots and stored at -20°C prior to analysis. Blood was collected by venipuncture into 10 mL tubes at St. Joseph’s Hospital outpatient laboratory. After waiting a minimum of 30 min for coagulation to occur, the tubes were centrifuged at 3000 rpm for 15 min. The serum was transferred into 1 mL cryovials (5 aliquots/subject) and kept at -20°C. All paired serum and saliva samples were collected within 30 min of each other. The saliva was always collected first. If, for any reason, the time between serum and saliva collection extended beyond 30 min, the results were not included.

### Saliva immunoassays

Saliva samples were thawed and centrifuged at 3000 rpm for 15 min, then submitted to assay using batch processing. For each hormone, each patient sample was analyzed in duplicate using a commercial high-sensitivity salivary enzyme immunoassay (Salimetrics LLC, an industry leader in salivary EIAs), following the manufacturer’s protocol. Cortisol, estradiol, estriol, progesterone, testosterone, and DHEA were analyzed. Repeat freeze-thaw cycles were avoided by using separately frozen aliquots for each hormone, which were thawed only as needed. Although most analytes are stable in saliva for ~5-7 days at room temperature [2,4], the measurement of some steroids can be altered by bacterial degradation after as little as 96 hr, even if stored at 4°C [51]. Therefore, the frozen aliquots were analyzed within only a few hours of thawing. Samples that exceeded the upper limit of the standard curve were re-analyzed after dilution. Table 1 shows the lower limit of detection for the 6 hormones and the intra-assay coefficients of variation.

**Table 1:** Inter- and intra-assay coefficients of variation and detection limits for the six salivary steroids

	**Intra CV%**	**Inter CV%**	**Detection limit**
Cortisol	3.5	5.1	0.08 nmol/L
Estriol	6.1	7.1	0.06 nmol/L
Estradiol	7.1	7.5	3.65 pmol/L
Progesterone	6.2	7.6	0.02 nmol/L
Testosterone	4.6	9.8	3.47 pmol/L
DHEA	5.6	8.2	0.02 nmol/L

### Serum radioimmunoassays

Estradiol, free estriol, testosterone, progesterone, and cortisol were assayed in duplicate using the Coat-A-Count^®^ radioimmunoassay (Diagnostic Products Corporation, Los Angeles, CA) according to the manufacturer’s instructions. DHEA was measured using MP Biomedicals DHEA kit 07D-229102. All hormones were batch processed. All assays employed a ^125^I label. Separate aliquots were used, thereby eliminating freeze-thaw cycles. Any samples that exceeded the upper limit of the standard curve were re-analyzed after dilution.

All saliva and serum assays were performed in the Psychiatric Research Laboratory at St. Joseph’s Healthcare in Hamilton, Ontario. The laboratory has over 10 years of experience using the Salimetrics kits.

### Statistical analysis

Data were analyzed with SPSS v.10 for Windows. Pearson correlation coefficients were used to compare the serum and saliva concentrations of each hormone.

## Results

### Salivary hormone concentrations

**Figure 1:**
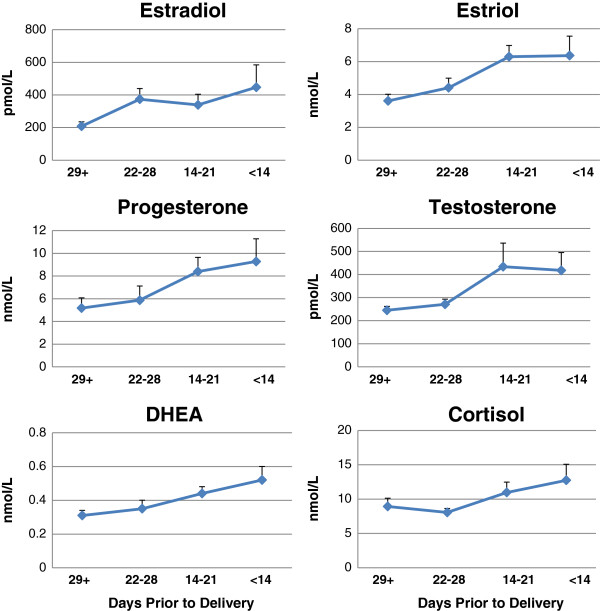
**Mean salivary steroid concentrations as a function of the number of days prior to parturition. **The women (*n *= 28) were tested between 32 and 38 weeks of gestation. Means are shown according to the number of days prior to delivery (< 14 days, 14–21 days, 22–28 days, 29+ days, *n=7*/group). Error bars represent the standard error of the mean.

Figure 1 shows mean concentrations for all 6 salivary steroids expressed as a function of the number of days prior to parturition (for further details, see Table 2). With the exception of estradiol, the concentrations of all hormones in saliva were significantly higher during the 3 weeks prior to delivery than at earlier time points. Polynomial contrasts confirmed a significant linear trend was present for salivary estriol, *F* (1, 24) = 9.72, *p* = 0.005; progesterone, *F* (1, 24) = 5.57, *p* = 0.027; testosterone, *F* (1, 24) = 4.90, *p* = 0.037, DHEA, *F* (1, 24) = 8.27, *p* = 0.008; and cortisol concentrations, *F* (1, 23) = 4.30, *p* = 0.049. One woman had atypically high estradiol in her saliva (>1000 pmol/L) at four weeks prior to delivery and as a result, the linear trend for estradiol was not significant.

**Table 2:** Salivary steroid concentrations in the antepartum women by days prior to delivery

		**Mean ± SEM**	**Median**	**Range**
**Estriol (nmol/L)**				
	< 14 days	6.37 ± 1.18	6.15	3.37 - 10.11
	14-21 days	6.30 ± 0.68	5.92	4.27 - 9.79
	22-28 days	4.41 ± 0.58	4.16	2.88 - 7.34
	> 28 days	3.61 ± 0.39	3.34	2.21 - 5.12
**Estradiol (pmol/L)**				
	< 14 days	447.23 ± 137.51	341	229 - 1128
	14-21 days	338.78 ± 65.87	289	67 - 653
	22-28 days	373.79 ± 65.39*	376	159 - 558
	> 28 days	208.54 ± 27.29	210	116 - 284
**Progesterone (nmol/L)**				
	< 14 days	9.28 ± 2.04	8.54	4.30 - 15.92
	14-21 days	8.39 ± 1.25	8.10	4.00 - 13.48
	22-28 days	5.87 ± 1.25	5.24	1.84 - 11.92
	> 28 days	5.18 ± 0.95	5.44	1.76 - 9.74
**Testosterone (pmol/L)**				
	< 14 days	418.44 ± 77.17	378	253 - 755
	14-21 days	434.54 ± 102.49	354	247 - 1129
	22-28 days	270.52 ± 22.65	281	180 - 341
	> 28 days	245.68 ± 17.24	257	183 - 301
**Cortisol (nmol/L)**				
	< 14 days	12.74 ± 2.32	10.34	7.92 - 22.35
	14-21 days	10.96 ± 1.50	9.74	6.41 - 17.75
	22-28 days	8.05 ± 0.57	8.50	4.78 - 9.58
	> 28 days	8.92 ± 1.28	8.48	5.45 - 12.60
**DHEA (nmol/L)**				
	< 14 days	0.52 ± 0.08	0.47	0.28 - 0.85
	14-21 days	0.44 ± 0.04	0.39	0.33 - 0.66
	22-28 days	0.35 ± 0.06	0.26	0.22 - 0.57
	> 28 days	0.32 ± 0.03	0.31	0.23 - 0.44

* = outlier removed, > 1000 pmol/L.

Patient’s depression status did not significantly influence the levels of any salivary analyte. This remained true after outlier removal, although when the estradiol outlier was removed, the mean estradiol concentration across all antepartum time points was found to be slightly lower in depressed than non-depressed women, *F* (1, 25) = 3.99, *p* = 0.057. Salivary cortisol was slightly higher in the depressed than non-depressed group after outlier removal, *F* (1, 25) = 3.47, *p* = 0.074, but neither hormone reached the criterion for significance. Depressed and non-depressed women did not differ in the timing of their antepartum specimen collection, *F* (1, 26) = 1.22, *p* = 0.279.

Table 3 shows the median and range of concentrations found for all 6 salivary steroids in the postpartum. Hormonal concentrations were not higher in the women tested beyond 19 weeks than in those tested between 4 and 16 weeks, therefore data are shown in Table 3 for all women combined. The mean number of weeks postpartum at the time of saliva collection was *M* = 11.9 and *M* = 25.4 wk in the two groups, respectively. For each of the gonadal steroids, the modal hormone concentration in the postpartum samples was below the limit of detection (LOD) of the saliva assays.

**Table 3:** Salivary steroid concentrations in postpartum women and percentage of samples that yielded detectable concentrations

	**Estradiol**	**Estriol**	**Progesterone**	**Testosterone**	**Cortisol**	**DHEA**
	**(pmol/L)**	**(nmol/L)**	**(nmol/L)**	**(pmol/L)**	**(nmol/L)**	**(nmol/L)**
Median:	8	ND	0.07	151	3.38	0.39
Mode:	ND	ND	ND	ND	1.57	0.68
Range:	ND - 28	ND - 0.08	ND - 0.22	ND - 418*	1.57 - 13.47	0.07 - 1.54*
%	81	2	60	81	100	100

ND = not detectable; % = percentage of samples that yielded concentrations falling above the detection limit (LOD) of the assay.

* = outlier removed, > 550 pmol (T) or > 3.50 nmol (DHEA).

### Serum hormone concentrations

As expected, serum hormone concentrations rose with advancing gestational weeks in the usual pattern, but individual differences in the hormone levels attained were substantial at all time points with considerable overlap between the different time points. Although estriol, *F* (1, 24) = 5.95, *p* = 0.022, progesterone, *F* (1, 24) = 6.35, *p* = 0.019, and to a lesser extent estradiol, *F* (1, 24) = 3.41, *p* = 0.077 showed a marked increase in serum concentration over the final antepartum weeks, this trend was not significant for DHEA, *F* (1, 24) = 1.93, *p* = 0.178, testosterone, *F* (1, 24) = 0.64, *p* = 0.431, or cortisol, *F* (1, 24) = 0.16, *p* = 0.691. As in the salivary results, mean serum estradiol was lower and cortisol was higher among women who met criteria for antepartum depression, but the differences were not statistically significant, *F* (1, 26) = 3.15, *p* = 0.088 and *F* (1, 26) = 2.90, *p* = 0.101, respectively.

### Serum-saliva correlations

Pearson correlations between saliva and serum hormone concentrations were calculated separately for the antepartum and postpartum data (see Table 4). There was no effect of patient depression status on the correlations observed between serum and saliva.

**Table 4:** Correlations between serum and saliva

	**Antepartum (n = 28)**	**Postpartum (n = 43)**		
	**r**	**r**_**OBS**_	**r**_**ZERO**_	**r**_**LOD/√2**_
Estradiol	.71**	.41*	.62**	.59**
Estriol	.71**	--	--	--
Progesterone	.65**	-.06	.48**	.40**
Testosterone	.48**^	.29^	.59**	.62**
Cortisol	.30	.60**	.60**	.60**
DHEA	.50**	.65**^	.65**	.65**

* p < .05, ** p < .01.

^ = outlier removed.

r_OBS _= observed correlation based only on samples with salivary concentrations > LOD; r_ZERO _and r_LOD/√2 _= correlation based on all 43 women with statistical correction for samples < LOD.

In the antepartum, correlations were statistically significant but only moderate in size for estradiol, estriol, and progesterone. For other hormones, the obtained correlations were low (r = 0.50 or below).

Postpartum, the correlations for all of the gonadal steroids were complicated by the large numbers of specimens falling below the detection threshold of the saliva assays. Complete data were available only for cortisol and DHEA (*n* = 43), and these hormones yielded the highest correlations (r = 0.60 and 0.65, respectively). A correlation could not be computed for estriol, as only one participant had an estriol concentration above threshold. Table 4 shows the correlation observed for each hormone (r_OBS_) based on only those women who had concentrations above the detectable threshold. Concentrations below the detection limit (LOD) of an assay are considered indeterminate, but if not included when calculating a correlation, such left-censoring can lead to a biased estimate of the association [52]. Accordingly, the same correlations were re-computed so as to include values below the limit, using 2 alternative data substitution methods: (i) by assigning a constant value of zero to any datapoints below the detection limit of each assay (denoted r_ZERO_ in Table 3) or (ii) assigning a value equal to LOD/√2, as recommended by Hornung and Reed [53] (r_LOD/√2_). Regardless of the method used, Table 4 shows that in the postpartum, the correlation between serum and salivary measurements was low for all hormones examined, with no correlation greater than about 0.60.

## Discussion

Saliva has been advocated as an important alternative to blood for the measurement of gonadal and adrenal steroids. Although the use of tandem mass spectrometry is increasing for steroid determinations in plasma and can provide an entire steroid profile in a single analysis, this technique has received only very limited adoption for salivary steroids. At present therefore, EIA or conventional RIA remain the major analytic methods in widespread use for salivary sampling. An impediment to the use of saliva by clinicians is a lack of reliable normative data regarding the concentration ranges to be expected under various physiological conditions. Pregnancy and the postpartum have been particularly understudied. Using paired serum and saliva specimens, we found that concentrations of all six steroids in maternal saliva exhibited a linear increase over the last few weeks of gestation. The ovarian steroids fell to early follicular values in the extended postpartum. In maternal serum, a steady increase in steroid concentrations with a peak near end of term is normally seen over the same time period [54,55] except for DHEA [54,56] and, in some studies, testosterone concentrations [54]. Relative to non-gravid values, DHEA in serum exhibits an increase that is greatest in the first and second trimesters and a decrease following parturition which is sustained for several months postpartum [56]. Bammann et al. [57] found that free testosterone, measured by equilibrium dialysis, was elevated in maternal plasma after gestational week 28 due to increased production rate, lending biological plausibility to the increase we observed in saliva. Saliva approximates the biologically active fraction of the circulating hormone [1,3].

Whether or not salivary concentrations increase during late gestation and whether any increase is proportional to the increase in total concentrations in serum, has not been established for most major steroids relevant to maternal physiology. In percentage terms, we observed the largest increases in late gestation for salivary estradiol and progesterone, while cortisol showed the smallest percent increase. Salivary cortisol levels resembled those seen using early morning sampling in women who are not pregnant (14.32 ± 9.1 nmol/L, n = 662, [58]). Early studies speculated that salivary cortisol may exhibit no increase during pregnancy in contrast to the marked rise found in serum, but our observation of modest elevation is consistent with reports of elevated basal cortisol late in gestation as measured by either RIA or EIA [59-61]. The concentrations detected in the present study were similar to these reports or other recent data [23]. The fact that pregnant women do not display overt signs of hypercortisolism may be explained by the relatively modest elevation in the bioavailable hormone combined with tissue refractoriness to raised cortisol levels [60,62].

Estriol is detectable in maternal serum in significant amounts only during pregnancy, when it is synthesized by the fetoplacental unit from adrenal precursors. Estriol concentrations rise exponentially in the 2 to 4 weeks before the onset of spontaneous labour. We found that estriol was quantitatively the most abundant free estrogen in antepartum saliva. Salivary estriol increased linearly during late gestation and the concentrations seen at all time points were within expected ranges based on other studies that have successfully measured estriol in saliva [19,20,22,63]. Suri et al. [63] used the Salimetrics EIA, the same EIA employed in the present work.

Aside from estriol and cortisol, normative data for other salivary steroids during gestation are significantly lacking. Available data often are based on a single gestational time point or else collapsed across large segments of the third trimester when hormone levels undergo continuous change, and are therefore difficult to interpret. In contrast to cortisol, by the final two weeks of gestation we found that progesterone and estradiol were present in saliva at mean concentrations 30 to 50 times their values under non-pregnant conditions, suggesting a substantial change in the bioavailable fraction of these two steroids. When compared with the limited data available, the concentrations we observed are similar to previous reports, both for salivary estradiol [40,64], and progesterone ([7,40]; but see [65]), although a progressive increase in salivary progesterone in late gestation was not detected by Lewis, Galvin, and Short [66]. We also found an increase in salivary testosterone over the last few weeks of pregnancy. In contrast, Laudat et al. [67] did not find evidence of increased testosterone relative to non-pregnant controls in saliva taken as late as 34 to 39 weeks gestation. However, the present data are consistent with reports that free testosterone in serum is increased about twofold in the third trimester [57,68].

Ten women in the present sample met DSM-IV criteria for depression during the antepartum. Although not significant given our sample size, there was a tendency for salivary estradiol to be lower and cortisol to be higher in women who were depressed. The same pattern was evident in serum. It would not be entirely surprising if cortisol levels were elevated in our depressed subgroup, as a few studies have found elevated cortisol in saliva [69,70], or urine [71,72] in antepartum depressed women, particularly women who have comorbid anxiety, although findings are conflicting [73,74]. Saliva may be a useful vehicle to study correlations between maternal hormones and mood because of its theoretical advantages and the practicality of repeat sampling. Further work is needed to verify the presence of hormonal alterations in antepartum depressed women and to identify any consequences for fetal development or long-term outcomes.

Postpartum concentrations also were analyzed. We found no significant difference in the salivary concentrations measured at 4 to 16 weeks postpartum *versus* beyond 19 weeks. Modal concentrations of the gonadal steroids fell below the analytical detection limit of the salivary EIAs in the postpartum. This is consistent with continuing anovulatory status in most of the women studied. Only 1–2 women in our dataset showed evidence of ovulatory activity within the study’s timeframe. Higher concentrations of ovarian steroids would be expected once menstrual cycling resumed. Only a handful of published studies have tried to quantify ovarian steroids in postpartum saliva. For progesterone and estradiol, our median concentrations closely resemble the three reports available [41,65], which found levels typical of the early follicular phase of the menstrual cycle at 5 and 6 weeks postpartum, respectively, and at 5 to 20 weeks postpartum [75] (see also [76]). With respect to estriol, Kundu et al. [8] estimated the estriol concentration in nongravid saliva to be < 0.11 nmol/L.

Because it contains largely the bioavailable fraction, expected steroid concentrations in saliva constitute only a small percentage of the total concentrations found in serum. Combined with low levels of ovarian activity in the first few months after delivery, our data suggest the postpartum period may challenge the detection limits of many existing salivary EIAs. From the standpoint of clinical chemistry some inevitable loss in assay precision is to be expected as the true concentrations in saliva approach the lowermost end of the calibration curve. Whether the detection of such low levels of hormone is biologically meaningful is an open question, but bioavailability has been of interest in the study of postpartum dysphoric mood changes [65]. Until other techniques become widely available, researchers who wish to quantify bioavailable hormone during the postpartum may need to utilize customized RIAs that can achieve lowered detection limits, and would be well-advised to collect larger sample volumes or multiple saliva samples to facilitate pooling and reduce measurement error attributable to pulsatile patterns of hormone secretion.

In contrast to the reproductive steroids, the adrenal corticoids, cortisol and DHEA, were present at quantifiable concentrations in the saliva of all women sampled postpartum. The return of the HPA axis to pregravid status is time-dependent, but the sensitivity of plasma cortisol to negative feedback regulation returns to normal by about 4 to 5 weeks postpartum [60,77]. Median salivary cortisol in the present study was similar to, or slightly lower than, the postpartum values seen by Harris et al. [65] at 6 to 8 weeks, and compare favorably with the concentrations reported by Tu et al. [75] at 5 to 20 weeks postpartum in primiparous mothers (see also [59]). Small differences across studies in salivary cortisol concentrations at matched times of day might reflect, among other variables, maternal parity.

Although salivary concentrations in the present work were within typical ranges both ante- and postpartum (within the limits of the literature available), the *correlations* we observed between serum and saliva were substantially below the magnitudes of the correlations for the same steroids ordinarily seen in non-gravid populations [13]. Low correspondence between serum and saliva was evident despite our use of widely utilized FDA-approved salivary EIAs and acceptable assay quality (Table 1). For estriol, a body of evidence currently exists that shows satisfactory correlations between serum and saliva during gestation (r’s = 0.75 to 0.98), although most studies have used RIA, not EIA techniques. For other hormones, there have been almost no published attempts to evaluate paired serum and saliva correlations in the ante- or postpartum. The low to moderate serum-saliva correlations in our antepartum data are consistent with the few existing reports from other labs (estradiol: r = 0.47-0.60 [40]; progesterone: 0.43-0.46 [40]; r = 0.88 [41]; cortisol: r = 0.55 to 0.77 [25,78]). Sample sizes in the present study were comparable to or even larger than most of these reports. One study reported a serum-saliva correlation of r = 0.92 for progesterone in pregnant women [7], but its reliability can be questioned as the correlation was calculated based on only 9 data points scattered throughout weeks 5 to 38 of gestation. Even fewer data currently exist based on postpartum sampling (progesterone and cortisol: r’s ≈ 0.50 [65]; cortisol: r = 0.42 - 0.60 [25]). Previous studies have used either mixed longitudinal (e.g., [22,41,78]) or cross-sectional data collection [7,19,25,40]. Mixed longitudinal designs have the potential to falsely inflate correlations by introducing dependencies among pairs of observations [79], but no obvious differences in the size of the correlations obtained are evident between the studies that have used cross-sectional *vs* longitudinal [20] or mixed longitudinal sampling. The low correlations found in the present work as well as these previous studies suggest that low correlations between serum and saliva measurements may not be atypical during late pregnancy and the early postpartum.

Low to moderate correlations could signify inaccuracies in the particular EIAs we chose to analyze saliva, but this seems unlikely given the acceptable performance of these same assays under other physiological conditions, and the comparability of the present concentration ranges in antepartum saliva to the concentrations identified in previous work that used either EIA or RIA. Conceivably, small differences in antibody specificity between the serum and saliva assays used here, although insignificant under ordinary circumstances, are amplified in importance under the high levels of circulating hormones present during late gestation and lead to reduced correlations to the extent that cross-reacting analytes change at different rates. EIA methods have, in fact, been criticized for their sometimes poor specificities [80]. The Salimetrics reagents used for quantification in the present study, however, have acceptably low (i.e., negligible) cross-reactivities with other major steroids present in antepartum saliva, with one exception. Indeed, while a correlation of only *r* = .30 was found for cortisol during late gestation in the present study (the lowest correlation we observed), the identical serum and saliva kits yielded a correlation of *r* = .96 in a set of patients screened for Cushing’s syndrome [81]. Although we think it unlikely that differences in antiserum specificity are responsible for the low correlations observed more generally, such a mechanism might explain the low correlation seen for testosterone, as the Salimetrics EIA shows higher cross-reactivity than the Coat-A-Count method for androstenedione and dihydrotestosterone [82], hormones that may plausibly be increased antepartum [55].

If not differences in antibody specificity, then what *does* account for the low correlations seen? In the *post*partum, the low correlations observed are likely to reflect the exceedingly low quantities of bioavailable hormone associated with ovarian quiescence, the consequent technical difficulty associated with the detection of such low quantities in saliva, and restriction in the *range* of concentration values to be expected in both serum and saliva, all factors that mitigate against finding high correlations. Under postpartum conditions, therefore, lower correlations are perhaps unsurprising. Low to moderate correlations seen *ante*partum are not easily attributed to technical limitations but, in contrast, could be physiologically significant--they may be a direct consequence of dissociations that occur between the total and bioavailable fractions in late gestation.

Complex changes in protein binding occur throughout pregnancy, but especially during the third trimester. By sequestering active hormone, rising titers of carrier proteins such as CBG and SHBG in the maternal circulation may serve to protect the mother from adverse consequences of the rise in fetal production of steroids [83]. Total concentrations of several steroids in maternal serum increase exponentially near end of term [55], but our data suggest that increases in saliva, at least over the timeframe we sampled, are potentially more linear and display a shallower rate of increase, consistent with the idea of only limited change in bioavailable hormone concentrations. We used a cross-sectional design and it is possible that increases in saliva concentrations would be sharper if sampled longitudinally, within an individual woman. However, serum concentrations in the present study were compatible with typical normative values for late gestation, and is it not obvious why any dampening attributable to the cross-sectional design would affect only the saliva. When computed across a range of gestational time points, correlations between serum and saliva thus may be attenuated by differential rates of change in the total *vs.* bioavailable compartments.

Dissociation between serum and saliva concentrations may be accentuated near end of term by competition from other steroids. For example, CBG increases during gestation, ensuring that unbound levels of cortisol are only mildly elevated in spite of dramatic increases in total cortisol concentration in serum. Because progesterone and cortisol bind to CBG with about equal affinity [84], the exponential rise in circulating progesterone that occurs in late gestation competes with cortisol for CBG binding sites, dynamically affecting the bioavailability of both hormones. Similarly, the sharp rise in estrogens near end of gestation may compete with the more modest rise in testosterone for available binding sites on slower changing quantities of SHBG [55], resulting in the displacement of some testosterone and increasing the free concentrations of both hormones. Collectively, such factors conspire to lower the correlations between saliva and the total hormone as measured in serum or plasma. If this hypothesis is correct, the implication is that serum and salivary assays do not provide redundant information during pregnancy and potentially may reveal complementary rather than interchangeable insights into maternal and fetal health.

## Conclusions

Saliva monitoring in pregnancy has the potential to advance our theoretical understanding of maternal physiology and to inform clinical practice. Some data suggest that salivary measures may have greater diagnostic relevance and predictive utility than conventional serum or plasma (e.g., [2,29]) because of the more direct estimation of biologically active hormone offered by saliva. Our study is among the first to investigate a range of salivary steroids during the third trimester and extended postpartum, and may help to address a significant gap in current literature by contributing toward the establishment of normative reference ranges for the salivary steroids examined. It will be desirable for future work to collect saliva from individual women at multiple time points during the late antepartum to verify the salivary profiles found in the present study. Low correlations between paired serum and saliva specimens collected less than 30 minutes apart suggest the two media may index different but overlapping fractions of the hormone present in the maternal circulation.

## Competing interests

The authors declare that they have no competing interests.

## Authors’ contributions

EH participated in the study design, performed statistical analyses, interpreted data, and drafted the manuscript. SDP recruited study participants, assisted in specimen collection, performed the assays, and assisted in data analysis and interpretation. CS and MS participated in study design and implementation and helped to draft the manuscript. MS coordinated the entire study and assigned clinical diagnoses to study participants. All authors have read and approved the final manuscript.
